# Exploring the use of micro-computed tomography (micro-CT) in the taxonomy of sea cucumbers: a case-study on the gravel sea cucumber *Neopentadactylamixta* (Östergren, 1898) (Echinodermata, Holothuroidea, Phyllophoridae)

**DOI:** 10.3897/zookeys.1054.67088

**Published:** 2021-08-04

**Authors:** Yves Samyn, Gontran Sonet, Cédric d’Udekem d’Acoz

**Affiliations:** 1 Scientific Service of Patrimony, Royal Belgian Institute of Natural Sciences, Vautierstraat 29, 1000, Brussels, Belgium Scientific Service of Patrimony, Royal Belgian Institute of Natural Sciences Brussels Belgium; 2 Joint Experimental Molecular Unit, Royal Belgian Institute of Natural Sciences, Vautierstraat 29, 1000, Brussels, Belgium Joint Experimental Molecular Unit, Royal Belgian Institute of Natural Sciences Brussels Belgium

**Keywords:** Integrative taxonomy, micro-CT, Normandy, scanning electron microscopy

## Abstract

Sea cucumber taxonomy and systematics has in the past heavily relied on gross external and internal anatomy, ossicle assemblage in different tissues, and molecular characterisation, with coloration, habitat, and geographical and bathymethric distribution also considered important parameters. In the present paper, we made these observations and techniques in detail and complemented them with the novel technique of micro-computed tomography of the calcareous ring. We investigated a single European species, the so-called gravel sea cucumber, *Neopentadactylamixta* (Östergren, 1898), using recently collected material from the Chausey Islands, Normandy, France. We redescribed the species, illustrated its ossicle assemblage through scanning electron microscopy, and visualised the calcareous ring through stacking photography and through micro-CT scanning. Additionally, a DNA fragment of 955 base pairs of the 18S ribosomal RNA gene was sequenced from one specimen, which showed a high similarity with the only sequence of *N.mixta* publicly available. We completed this integrative study by providing a detailed distribution of the occurrence of *N.mixta* based on published, verifiable accounts.

## Introduction

Recently, during a leisure expedition, Mr Francis Kerckhof of the Royal Belgian Institute of Natural Sciences collected four specimens of sea cucumbers on a beach in the Chausey Archipelago, Normandy, France. Collecting was done during a low, spring tide, between rocks, on coarse/gravelly sand mixed with shell (fragments), which is characteristic of a wave-beaten environment.

The species was identified as *Neopentadactylamixta* (Östergren, 1898), the gravel-sea cucumber, based on ossicles and the structure of the calcareous ring.

Examination of the calcareous ring, one of the key characters differentiating (sub)families in the order Dendrochirotida Grube, 1840, has in the past been made through intrusive and partly destructive dissections. Micro-computed tomography (i.e., micro-CT or µCT) provides an alternative, non-destructive method to decipher the structure of the calcareous ring. The goal of this paper is to evaluate the efficacy of this method.

Improved methods to study calcareous rings are needed in comparative taxonomic research. Smirnov (2012: 806), after a detailed and well-considered reflection of nearly 200 years of holothuroid classification noted: “the morphology of the calcareous ring is very important, although it is very difficult to describe its morphology and even more difficult to characterize features distinguishing the orders”. Other authors, e.g., Cherbonnier (1988) and Thandar (1989), also made extensive use of the structure of the calcareous ring to distinguish dendrochirotids at family and subfamily levels. Study of the calcareous ring has generally been accomplished through dissection, except for some sea cucumbers with a translucent body (e.g., *Epitomaptasimentalae*Solis-Marin et al., 2019) or those that readily expel their calcareous ring upon collection (e.g., species of the genus *Massinium* Samyn & Thandar, 2003).

In a broad phylogenetic study of Holothuroidea, Miller et al. (2017) noted that some morphological characters in holothuroid systematics are subject to homoplasy. More detailed anatomical studies, such as non-invasive micro-CT scanning of the calcareous ring and also, to a certain extent, other hard substances such as ossicles, allow a more in-depth appraisal of these structures and may offer resolution of apparently homoplastic characters.

Here we combine a traditional description of external and internal morphology and anatomy, with a *de novo* illustration of ossicle assemblages through SEM (the only and last detailed illustrations for *N.mixta* was provided by decades ago by Féral 1979), a genetic characterization through the sequencing of 955 base pairs (bp) of the 18S ribosomal gene that we compared with the only available 18S DNA sequence available for this species (Lacey et al. 2005), and a visualization of the calcareous ring through micro-CT as well as focus-stacked photography. This threefold approach is an attempt to arrive at an innovative approach to describe and classify sea cucumbers in a more modern and integrative way.

## Materials and methods

On 20 April 2019, four specimens of *Neopentadactylamixta* were collected at extreme low tide on the Plage de la Grande Grève, (48°52.5'N, 1°50.8'W) on the Grande Île of the Chausey Archipelago, France. The specimens were relaxed in a solution of ±5% MgCl_2_·6H_2_0 prior to fixation with 70–75% ethanol for 1 day, whereafter they were preserved in 70% ethanol denatured with diethyl ether for another 2 days before being placed in 70% buffered diethyl ether ethanol for permanent storage.

Ossicles from one out of the four specimens were prepared for light and scanning electron microscopy (SEM) by dissolving small pieces of dorsal and ventral body wall, tube feet, papillae, tentacles, and longitudinal muscle in household bleach, carefully rinsed with distilled water (Samyn et al. 2006) and observed through the microscope; no permanent slides were made. For SEM, ossicles were dried and mounted on aluminium stubs, coated with gold in a sputter coater, and observed with a FEI/Philips XL30 ESEM TMP Scanning Electron Microscope.

Micro-CT scanning was done with a RX EasyTom (RX Solutions, Chavanod, France; http://www.rxsolutions.fr), with an aluminium filter at the Royal Belgian Institute of Natural Sciences in Brussels, Belgium. No contrast agent was used on the specimen studied. For the 3D visualisation of the specimen (Fig. 2A) images were generated at a voltage of 88 kV and a current of 272 μA, with a set frame rate of 3.25 and 5 average frames per image. This generated 1824 images and a voxel size of 28.63 μm. For the calcareous ring *per se* (Fig. 2B, C), images were generated at a voltage of 71 kV and a current of 412 μA, with a set frame rate of 4 and 5 average frames per image. This generated 2016 images and a voxel size of 23.90 μm. Reconstructions were performed using X-Act software from RX Solutions. Segmentation, visualization, and analysis were performed using Dragonfly software for Windows (Object Research Systems (ORS) Inc., Montreal, Canada, 2020; software available at http://www.theobjects.com/dragonfly). 3D mesh files are available on https://sketchfab.com/3d-models/be-rbins-hol-1736-neopentadactyla-mixta-b013d76558234a84a4b6907132eff93d.

The stacked photo reconstruction of the calcareous ring and its surrounding anatomical structures was done with a reflex Canon EOS 6D Mark II equipped with a Canon MP-E 65mm macro lens. This set-up was fixed on a Cognisys StackShot Macro Rail which is guided with Helicon Remote software. Photo-stacking on 30 different pictures was done with Zerene Stacker Software.

DNA was extracted from a piece of body wall using the NucleoSpin Tissue Kit following the manufacturer’s protocol (Macherey-Nagel, Germany). Even though other DNA fragments are known to provide better resolution for DNA-based species identification, a fragment of 955 bp of the 18S ribosomal RNA gene was targeted because it is currently the only DNA sequence publicly available for *N.mixta*. The 18S ribosomal RNA gene was amplified using a nested polymerase chain reaction (PCR) protocol based on the primers and the conditions described by Lacey et al. (2005). The PCRs were prepared in volumes of 25 µl containing 1 μl of DNA template, 0.03 U/μl of Platinum Taq DNA Polymerase (Life Technologies, USA), 1X PCR buffer, 0.2 mM dNTPs, 0.4 μM of each primer, and 1.5 mM MgCl_2_. The first PCR was performed with primers EC-2F (AYCTGGTTGATYYTGCCAG) and WN-1708R (TGATCCATCTGCAGGTTCACCT) with the following thermal profile: one step at 94 °C for 3 min, then 40 cycles at 94 °C for 45 s, 55 °C for 45 s and 72 °C for 120 s, and a final step at 72 °C for 7 min. The second PCR was performed with primers 421-F (AAACGGCTACCACATCCAAG) and 1482-R (AGGGCATCACAGACCTGTTA), using 1 µl of the product of the first PCR as template DNA and with the following thermal profile: one step at 94 °C for 3 min, then 40 cycles at 94 °C for 30 s, 53 °C for 30 s and 72 °C for 120 s, and a final step at 72 °C for 7 min. PCR products were visualized on a 1.2% agarose gel after electrophoresis at 100V and purified using the ExoSAP procedure (Exonuclease I – Shrimp Alkaline Phosphatase from ThermoFisher, USA). PCR products were sequenced bi-directionally on an ABI automated capillary sequencer using the BigDye v. 3.1 chemistry following the manufacturer’s instructions (Life Technologies, USA). DNA chromatograms were checked, trimmed and assembled using CodonCode Aligner v. 8.0.2 (CodonCode Corp., Centerville, Massachusetts). Consensus sequence was compared to public records using the Basic Local Alignment Search Tool (BLAST) (Altschul et al. 1990) of the National Center for Biotechnology Information, U.S. National Library of Medicine (NCBI) and submitted to GenBank (accession number MW522513).

## Results

### Phyllophoridae Östergren, 1907

***Neopentadactyla*** **Deichmann, 1944**

#### Neopentadactyla mixta

Taxon classificationAnimaliaDendrochirotidaPhyllophoridae

(Östergren, 1898)

4BF41965-8EAD-535A-8752-025BC9763C11

Figures 1A–L , 2A–D

Pseudocucumis mixta Östergren, 1898: 104, 105, 135, fig. 3 (p. 109).
Pseudocucumis mixta : Bedford 1898: 843 (discussion); Östergren 1902: 24, note 1; Östergren 1904: 659; Östergren 1906: 1–24, figs 1–3; Ohshima 1912 54, 59 (discussion); Lieberkind 1929: 14; Massy 1920: 52; Koehler 1921: 168, fig. 124; Mortensen 1924: 236, fig. 116; Koehler 1927: 195, pl. 16, fig 16; Engel 1933: 33–34 (distribution); Cherbonnier 1952: 570, figs 1, 2; Könnecker and Keegan 1973: 157–162 (*in situ* pictures).
Pseudocucumis Cuenoti Koehler & Vaney, 1905: 395, figs 1–6.
Neopentadactyla mixta : Deichmann, 1944: 736; Heding and Panning 1954: 186, fig. 91; Féral 1979: 111, figs A–L; Féral 1980: 42, figs 1, 2; Smith 1983: 301, figs 1–4; Wood 1988: 143, 151 (drawing), 179; Moyse and Tyler 1990: 866 (key), 869, fig. 15.14 (upper left); Hansen and McKenzie 1991: 123 (lectotype and 3 paralectotypes), fig. 126–140; McKenzie 1991: 126, fig. 1; 132, fig. 3a–d; Picton 1993: 78 (colour photograph); McKenzie 1997: 274; Southward and Campbell 2006: 224, fig. 201.

##### Status and location of types.

Museum of Evolution, Uppsala University, Sweden (UPSZTY 2346): lectotype (249a); 2 paralectotypes (249b) (designated by McKenzie in 1990 according to the database of the Upsala Museum). McKenzie (1991) stated that he did not designate a lectotype and paralectotypes. In another publication Hansen and McKenzie (1991: 123) did designate and describe the lectotype (“Typsamlingen Nr. 249a”) from the four syntypes as present in the Uppsala Museum’s collection; the three remaining syntypes therefore become paralectotypes. Hansen and McKenzie (1991: 123) stated that they have maintained the original division of the four syntypes over two jars (249a and 249b), “each containing two specimens one of which was dissected, the other intact”. According to Hansen and McKenzie (1991), the lectotype is thus together with one of the paralectotype in Jar 249a, while the other two paralectotypes are in jar 249b. We did not revisit this type series.

##### Type locality.

W. Norway (most likely Molde, i.e., about 62°45'N, 7°14'E).

##### Material examined.

RBINS I.G. 33990, HOL.1736 (4 specimens plus SEM stubs: I.G. 33990/HOL.1736/1-8).

##### Description

**Figure 1.**
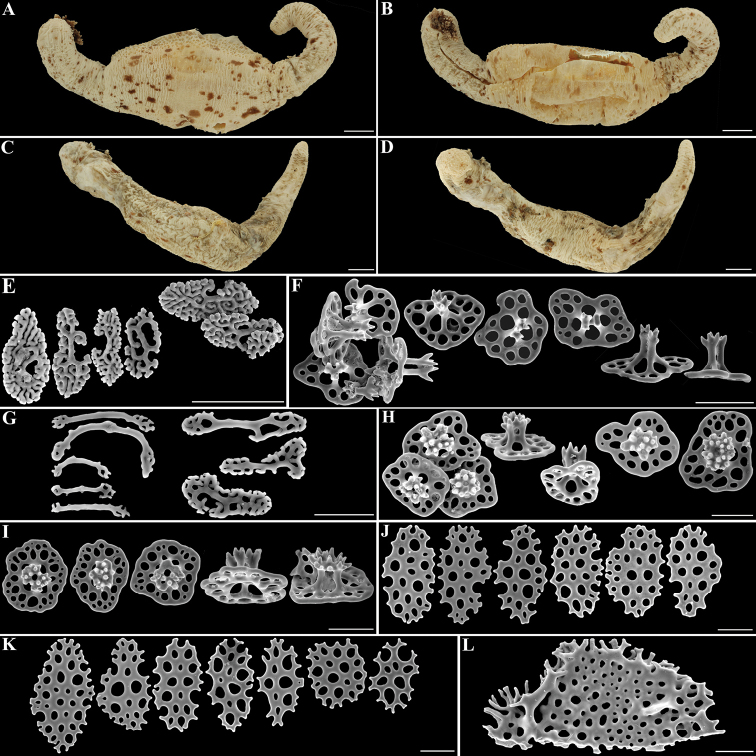
*Neopentadactylamixta* (Östergren, 1898) **A** focus-stacked view of the dorsal-lateral view of dissected specimen **B** focus-stacked view of the ventral–lateral view of dissected specimen **C** focus-stacked view of the dorsal–lateral view of a non-dissected specimen **D** focus-stacked view of the ventral–lateral view of a non-dissected specimen **E**SEM view of the rosettes from the shaft of a tentacle **F**SEM view of the 2-pillared tables from the introvert **G**SEM view of the rods and rosettes from a tentacle tip **H**SEM view of the 4-pillared tables from the dorsal body wall **I**SEM view of the 4-pillared tables from the ventral body wall **J**SEM view of the plates from the dorsal tube feet **K**SEM view of the plates from the ventral tube feet **L**SEM view of half of an end-plate from a ventral tube foot. Scale bars: 1 cm (**A–D**); 50 μm (**E–L**).

**Figure 2.**
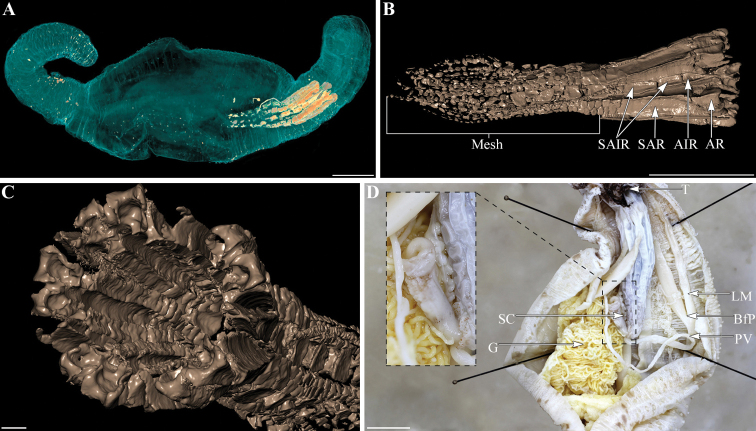
*Neopentadactylamixta* (Östergren, 1898) **A** micro-CT scan visualizing the position of the calcareous ring **B** lateral view with micro-CT imaging of the anterior part of the calcareous ring (AR: most anterior radial piece; AIR: most anterior interradial pieces; SAR: subsequent anterior radial pieces; SAIR: subsequent interradial anterior pieces; Mesh: meshwork of radial and interradial median to distal pieces) **C** oblique view with micro-CT imaging showing a guttered internal side of the calcareous ring **D** focus-stacked view of the calcareous ring and associated structures (T: tentacles; LM: longitudinal muscle with bifurcation point (BfP); PV: Polian vesicle: SC: stone canal). Scale bars: 1 cm (**A–D**).

**(based on material examined).** Body elongate, with central part inflated and anterior and posterior ends more narrow. Length of fixed specimens 50–155 mm (measured along the dorsal face); diameter 30–80 mm at mid-body, 21–43 mm anteriorly and 12–25 mm posteriorly. Color in alcohol after a short period of preservation same as color in life: body light beige, with irregular, brownish spots and patches (Fig. 1A, D); patches larger ventrally. Tentacles with their shaft light beige and the branches brownish with beige tips. According to McKenzie (1991), 10 large, five small, and five intermediate sized tentacles can be observed in live specimens. Insufficient relaxation of the specimens at hand made it impossible to observe the exact position of the tentacles in the specimens under study. Tube feet predominantly in the radii, in irregular double series anteriorly and posteriorly and in up to six rows, spreading into the interradii ventrally. Tube feet light beige. Body wall gritty to the touch. Body wall thin mid-body, thicker anteriorly and distally, possibly an artefact of fixation. Longitudinal muscles visible through the body wall where the skin is thinnest. Longitudinal muscles thick, undivided, and attached to the body wall along their entire width. Retractor muscles arise about 1/3 of the body length from anterior end, attaching to the radial section of the long and thin tubular calcareous ring, which itself is about 1/4 of the body length (Fig. 2A). Calcareous ring with five radial and five interradial parts (Fig. 2B). The radial parts are anteriorly notched, with 1–3 larger plates anteriorly and an irregular meshwork of smaller plates posteriorly. The interradial parts are anteriorly pointed, have 3 or 4 larger plates anteriorly and an irregular meshwork of smaller plates posteriorly (Fig. 2B). Calcareous ring embedded in a thin layer of tissue, with the calcified elements connected by connective tissue. (Fig. 2D). No clear posterior ending visible at the end of the comet-shaped, calcareous ring (Fig. 2A). Internal surfaces of especially the radial pieces are guttered (Fig. 2C). Single, very long Polian vesicle; single curled stone canal embedded in the dorsal mesentery (Fig. 2D).

Tentacle shafts with irregular, complex rosettes, 30–45 μm long and 15–25 μm wide (Fig. 1E); tentacle tips with straight to curved, terminally perforated rods, 30–55 µm long and rosettes similar to those of the tentacle shafts (Fig. 1G); introvert with 2-pillared tables only, disc smooth, 65–80 µm in diameter, irregular in outline, perforated by four central holes and a variable number of irregularly peripheral holes; pillars 40–65 μm high, with single cross-beam, ending in a narrow, sparsely-spined crown (Fig. 1F); dorsal and ventral body wall with 4-pillared tables, 80–100 μm in diameter, smooth rim, irregular in outline, perforated by four central holes and a variable number of peripheral holes arranged in multiple irregular circles; pillars 60–76 μm high, with single cross-beam, ending in a narrow, spined crown (Fig. 1H, I). Dorsal and ventral tube feet with plates, 64–95 μm long and 40–55 μm wide; endplate ±200 μm in diameter, with uneven sized holes and with some relief (Fig. 1L). Contrary to Féral’s (1980) observation, no tables could be found in the tube feet. Longitudinal muscles devoid of ossicles.

##### Distribution.

North and West European coasts: Molde, West Norway (Ötsergren 1898); Arcachon, Bréhat Island, Wimereux, Roscoff, Chausey Islands, France (Koehler and Vaney 1905; Koehler 1921; Cabioch 1965; Féral 1979, 1980; this study); Northern British Islands, Faroe Islands (Östergren 1906; Lieberkind 1929); Tatihou Island, Normandy, France (Östergren 1906), Ireland (Massy 1920; Könnecker and Keegan 1973; Féral 1979); Denmark (Mortensen 1924); Swedish and Norwegian waters (Hansen and McKenzie 1991).

##### Bathymetric range.

Intertidal (present study) to 200 m depth (Southward and Campbell 2006).

##### Ecology.

*Neopentadactylamixta* is most frequently found in maerl beds and coarse shell gravels with fairly strong tidal streams. It is a gregarious species, which may occur in densities of up to 297 individuals/m^2^ (Könnecker and Keegan 1973; McKenzie 1991; Picton 1993). Könnecker and Keegan (1973) reported *N.mixta* to be a rheophilic suspension feeder with diurnal feeding rhythm. Smith and Keegan (1985) demonstrated that *N.mixta* individuals on the west coast of Ireland stop their suspension feeding from autumn to spring and retire to depths of 30–60 cm in the coarse sediments during that period. As with other phyllophorids (e.g., *Massiniummaculosum* Samyn & Thandar, 2003), this species exposes only its tentacle crown and part of its introvert and the tip of its anus.

The sediment from the beach where the studied specimens were collected consisted of coarse, gravelly sand, characteristic for a wave-beaten environment and harboured a very rich and diverse fauna of other burrowing taxa (Bivalvia, Polychaeta, Sipuncula, Nemertea, etc.)

##### Systematics.

The DNA sequence retrieved from GenBank (http://www.ncbi.nlm.nih.gov) most similar to the 18S sequence obtained here was labelled as *Neopentadactylamixta* (accession number AY133482). Its similarity with our sequence is 99.16%. This sequence is currently the only DNA sequences available online for this species. The next most similar public DNA records were from *Phyrellamookiei* Michonneau & Paulay, 2014 (KX856842, 97.18%), a phyllophorid, and *Afrocucumisafricana* (Semper, 1867) (KX856841, 97.18%), a sclerodactylid. The high DNA similarity with *N.mixta* supports the morphological identification of the specimen studied here as *N.mixta*, as a species belonging to Phyllophoridae. This DNA-based result is backed up by its ecology, the gross morphology of the specimens, the structure of the calcareous ring, and the ossicle assemblage. However, the high DNA similarity with *A.africana* is troubling, as *Afrocucumis* Deichmann, 1944 is characterized by a very different “skeletal structure”: the calcareous ring has its radial pieces with two short, unsegmented, projections; the interradial pieces are without posterior projections; and the body wall holds large, 310–400 μm wide lenticular plates (Massin 1996). In *N.mixta*, no lenticular plates are present, and the calcareous ring is a much more complex structure (see the description above).

##### Deposition of images.

SEM images of ossicles and a focus-stacked image of the calcareous ring has been put on the Royal Belgian Institute of Natural Sciences “Virtual Collections” website at http://virtualcollections.naturalsciences.be/virtual-collections/recent-invertebrates/echinodermata#c4=N&b_start=0.

3D mesh files have been put on https://sketchfab.com/3d-models/be-rbins-hol-1736-neopentadactyla-mixta-b013d76558234a84a4b6907132eff93d.

## Discussion

*Neopentadactylamixta* was originally attributed to the monotypic genus *Pseudocucumis* Ludwig, 1875 by Östergren (1898). However, as correctly noted by Deichmann (1944), *Pseudocucumis* was established to accommodate *Cucumariaacicula* Semper, 1867 because of its particular ossicle assemblage (i.a. tables with reduced disc and fused pillars ending in a narrow crown). *Cucumariaacicula*, however, was referred to the genus *Urodemas* Selenka, 1867 (now considered a junior synonym of *Cladolabes* Brandt, 1835) by Deichmann (1944), making *Pseudocucumis* the junior synonym of *Urodemas* and *Cladolabes*. Deichmann (1944) proposed the generic replacement name *Neopentadactyla* Deichmann, 1944 to accommodate *Pseudocucumismixta*. *Neopentadactyla* has remained monotypic ever since (Heding and Panning 1954).

Heding and Panning (1954) divided the Phyllophoridae into several new subfamilies and put *Neopentadactyla* in Semperiellinae Heding & Panning, 1954. Since then the systematics of the Dendrochirotida has changed. In an attempt to bring order to the higher systematics of dendrochirote holothuroids, Pawson and Fell (1965) proposed two new families (Placothuriidae and Paracucumidae) and retained the Phyllophoridae as one of the valid families in the order. They diagnosed the Phyllophoridae as those dendrochirotid sea cucumbers with a calcareous ring composed of a mosaic of small pieces, unlike Heding and Panning (1954) who diagnosed Phyllophoridae as those dendrochirotids that present more than 10 tentacles, do not present a well-defined ventral sole, and predominantly present tables or derivatives thereof in their body wall. Thandar (1990) complemented Pawson and Fell’s (1954) diagnosis with the description of the ossicle assemblage and the number of tentacles.

Micro-CT scans have been extensively used by one of us (CUA) for imaging hard structures in echinoderms, including calcareous rings of Holothuroidea. Results have been variable but sometimes they have revealed structures that could not be otherwise visualized. Micro-CT images have advantages and disadvantages compared to photography. The main advantage of this rather new technique is the non-destructive approach (especially important when studying rare species or type specimens) and its theoretical capacity to separate hard structures from soft tissues. The structures can also be easily rotated in all orientations, allowing not only for snapshots in optimal positions, but also for visualizing features in their original position and this in three dimensions. However, in practice, there is often a gradient of opacity to X-rays between genuine soft tissues and fully ossified structures. Therefore, it is sometimes difficult to decide of an optimal image setting and misleading image artefacts can appear if too much material of medium X-ray density is digitally removed. Problems of this nature were met with *N.mixta*, without being too serious. Another disadvantage is the usually rather low resolution of the images, where the surface details can be erased by excessive smoothing. Scanning and editing scans are time consuming and sometimes expensive operations, which require the work of an experienced operator. In the case of *N.mixta*, micro-CT scans provided images which, while not absolutely perfect, allowed for a more detailed analysis and interpretation of the structure of the calcareous ring. However, they proved insufficient for illustrating the ossicle assemblage. Thus, ossicles were imaged using more traditional scanning electron microscopy.

In conclusion, micro-CT scanning is expensive and demands experienced operators with knowledge of the anatomical structures to be visualized. Here, we applied this technique to the calcareous ring, a key taxonomic character within the Dendrochirotida/Phyllophoridae. Imaging of the ossicle assemblage through micro-CT scanning proved insufficient and SEM is here preferred, both to analyse the structure and the dimensions of the ossicle assemblage. Species identification could be verified by comparison with the only other DNA sequence available in BOLD.

## Supplementary Material

XML Treatment for Neopentadactyla mixta
